# 3-(1,2-Di-*p*-tolyl­vin­yl)-2-methyl-1*H*-indole

**DOI:** 10.1107/S1600536812021617

**Published:** 2012-05-19

**Authors:** R. Senthamizhselvi, G. Bhaskar, P. R. Seshadri, P. T. Perumal, K. Illangovan

**Affiliations:** a Department of Chemistry, Easwari Engineering College, Ramapuram, Chennai 600 089, India; b Organic Chemistry Division, Central Leather Research Institute, Chennai 600 020, India; c Post Graduate and Research Department of Physics, Agurchand Manmull Jain College, Chennai 600 114, India; d Post Graduate and Research Department of Physics, RKM Vivekananda College, Chennai 600 004, India

## Abstract

In the title compound, C_25_H_23_N, the indole unit makes a dihedral angles of 79.03 (5) and 61.82 (4)° with the benzene rings. No classical hydrogen bonds are found in the crystal structure.

## Related literature

For the biological activity of indole derivatives, see: Olgen & Coban (2003 ▶); Joshi & Chand (1982 ▶).
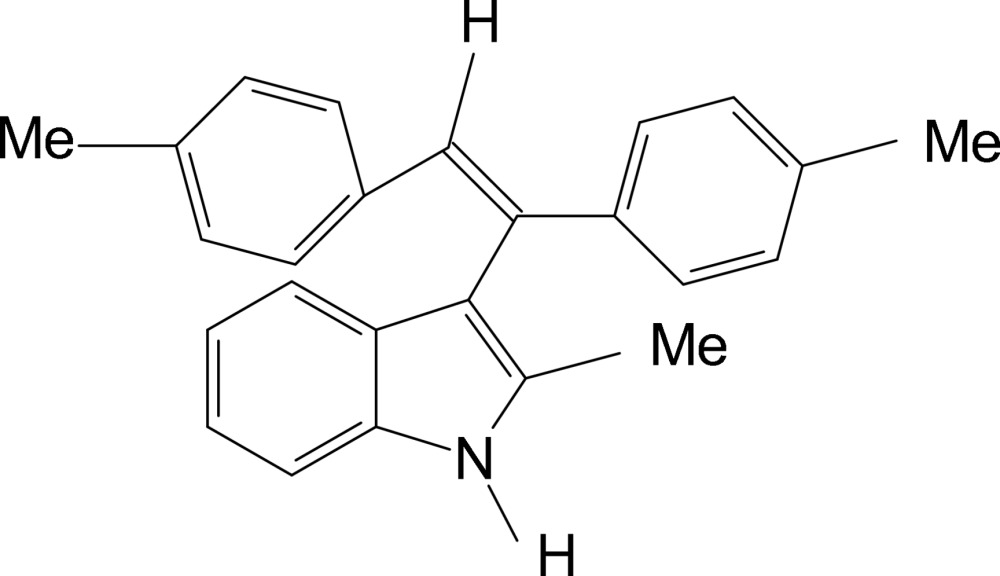


## Experimental

### 

#### Crystal data

C_25_H_23_N
*M* *_r_* = 337.44
Monoclinic, 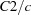

*a* = 25.684 (6) Å
*b* = 9.911 (2) Å
*c* = 16.739 (4) Å
β = 112.646 (5)°
*V* = 3932.7 (16) Å^3^
*Z* = 8
Mo *K*α radiation
μ = 0.07 mm^−1^
*T* = 298 K
0.20 × 0.18 × 0.15 mm

#### Data collection

Bruker SMART APEXII area-detector diffractometer
17685 measured reflections
4931 independent reflections
3008 reflections with *I* > 2σ(*I*)
*R*_int_ = 0.041

#### Refinement

*R*[*F* ^2^ > 2σ(*F* ^2^)] = 0.050
*wR*(*F* ^2^) = 0.159
*S* = 1.02
4931 reflections
238 parameters
H-atom parameters constrained
Δρ_max_ = 0.18 e Å^−3^
Δρ_min_ = −0.14 e Å^−3^

### 

Data collection: *APEX2* (Bruker, 2008 ▶); cell refinement: *SAINT* (Bruker, 2008 ▶); data reduction: *SAINT*; program(s) used to solve structure: *SHELXS97* (Sheldrick, 2008 ▶); program(s) used to refine structure: *SHELXL97* (Sheldrick, 2008 ▶); molecular graphics: *ORTEP-3* (Farrugia, 1997 ▶) and *PLATON* (Spek, 2009 ▶); software used to prepare material for publication: *SHELXL97* (Sheldrick, 2008 ▶), *PLATON* and *publCIF* (Westrip, 2010 ▶).

## Supplementary Material

Crystal structure: contains datablock(s) I, global. DOI: 10.1107/S1600536812021617/bt5910sup1.cif

Structure factors: contains datablock(s) I. DOI: 10.1107/S1600536812021617/bt5910Isup2.hkl

Supplementary material file. DOI: 10.1107/S1600536812021617/bt5910Isup3.cml

Additional supplementary materials:  crystallographic information; 3D view; checkCIF report

## Figures and Tables

**Fig. 1.**
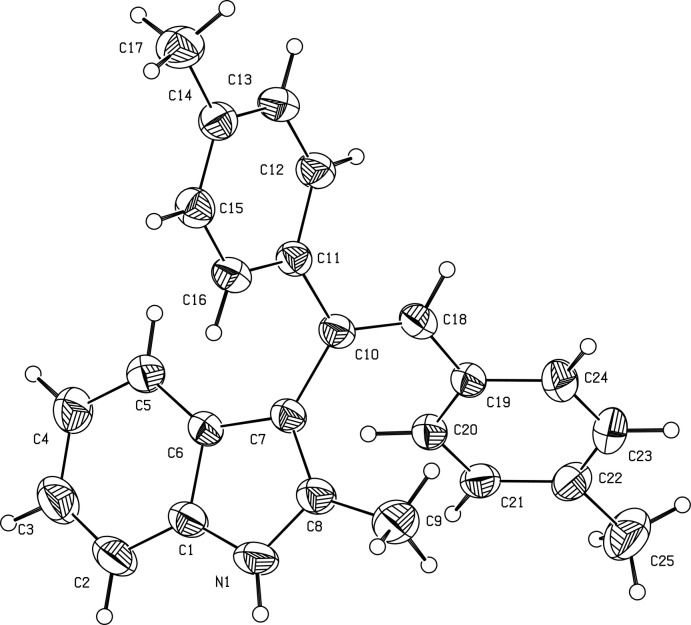
The molecular structure of the title compound, showing the atom-numbering scheme. Displacement ellipsoids are drawn at the 30% probability level.

## supplementary crystallographic information

### Comment

Indole derivatives exhibit anti-oxidant (Olgen & Coban, 2003), anti-bacterial and fungicidal (Joshi & Chand, 1982) activities. Against this background, the title compound was chosen for X-ray structure analysis (Fig. 1).

The phenyl rings C11—C16 and C19—C24 form dihedral angles with the indole ring system of 79.03 (5)° and 61.82 (4)°, respectively.

The sum of the bond angles around N1 [359.99 (3)°] indicates *sp*^2^ hybridization.

No classical hydrogen bonds are found in the crystal structure.

### Experimental

A mixture of di- *p*-tolylacetylene (3.0 mmol), 2-methyl indole (2.0 mmol), and indium bromide (0.2 mmol) in toluene (4 ml) was stirred at 110 oC for the 120 min t s time. After completion of the reaction as indicated by TLC, the reaction mixture was diluted with water and extracted with ethyl acetate. The combined organic layers were dried over anhydrous Na2SO4, concentrated *in vacuo*, and purified by column chromatography on silica gel (Merck, 100–200 mesh) to afford a mixture of E and *Z* (>99%) isomers. These major isomers were separated by crystallization using ethyl acetate/petroleum ether.

### Refinement

Hydrogen atoms were positioned geometrically and allowed to ride on their parent atoms, with N—H = 0.86 Å or C—H = 0.93 - 0.97 Å and *U*_iso_(H) = 1.5*U*_eq_(C_methyl_) or 1.2 *U*_eq_(C,N).

### Crystal data

**Table:**

C_25_H_23_N	*F*(000) = 1440
*M**_r_* = 337.44	*D*_x_ = 1.140 Mg m^−^^3^
Monoclinic, *C*2/*c*	Mo *K*α radiation, λ = 0.71073 Å
Hall symbol: -C 2yc	Cell parameters from 4931 reflections
*a* = 25.684 (6) Å	θ = 1.7–28.5°
*b* = 9.911 (2) Å	µ = 0.07 mm^−^^1^
*c* = 16.739 (4) Å	*T* = 298 K
β = 112.646 (5)°	Block, colourless
*V* = 3932.7 (16) Å^3^	0.20 × 0.18 × 0.15 mm
*Z* = 8	

### Data collection

**Table:**

Bruker SMART APEXII area-detector diffractometer	3008 reflections with *I* > 2σ(*I*)
Radiation source: fine-focus sealed tube	*R*_int_ = 0.041
Graphite monochromator	θ_max_ = 28.5°, θ_min_ = 1.7°
ω and φ scans	*h* = −30→34
17685 measured reflections	*k* = −13→7
4931 independent reflections	*l* = −22→21

### Refinement

**Table:**

Refinement on *F*^2^	Primary atom site location: structure-invariant direct methods
Least-squares matrix: full	Secondary atom site location: difference Fourier map
*R*[*F*^2^ > 2σ(*F*^2^)] = 0.050	Hydrogen site location: inferred from neighbouring sites
*wR*(*F*^2^) = 0.159	H-atom parameters constrained
*S* = 1.02	*w* = 1/[σ^2^(*F*_o_^2^) + (0.0719*P*)^2^ + 0.9275*P*] where *P* = (*F*_o_^2^ + 2*F*_c_^2^)/3
4931 reflections	(Δ/σ)_max_ = 0.004
238 parameters	Δρ_max_ = 0.18 e Å^−^^3^
0 restraints	Δρ_min_ = −0.14 e Å^−^^3^

### Special details

**Table:**

Geometry. All e.s.d.'s (except the e.s.d. in the dihedral angle between two l.s. planes) are estimated using the full covariance matrix. The cell e.s.d.'s are taken into account individually in the estimation of e.s.d.'s in distances, angles and torsion angles; correlations between e.s.d.'s in cell parameters are only used when they are defined by crystal symmetry. An approximate (isotropic) treatment of cell e.s.d.'s is used for estimating e.s.d.'s involving l.s. planes.
Refinement. Refinement of *F*^2^ against ALL reflections. The weighted *R*-factor *wR* and goodness of fit *S* are based on *F*^2^, conventional *R*-factors *R* are based on *F*, with *F* set to zero for negative *F*^2^. The threshold expression of *F*^2^ > σ(*F*^2^) is used only for calculating *R*-factors(gt) *etc*. and is not relevant to the choice of reflections for refinement. *R*-factors based on *F*^2^ are statistically about twice as large as those based on *F*, and *R*- factors based on ALL data will be even larger.

### Fractional atomic coordinates and isotropic or equivalent isotropic displacement parameters (Å^2^)

**Table:**

	*x*	*y*	*z*	*U*_iso_*/*U*_eq_	
C1	0.23355 (7)	0.99325 (17)	0.37403 (11)	0.0635 (4)	
C2	0.25810 (8)	1.1152 (2)	0.40950 (14)	0.0822 (6)	
H2	0.2939	1.1187	0.4532	0.099*	
C3	0.22776 (9)	1.2299 (2)	0.37794 (15)	0.0880 (6)	
H3	0.2432	1.3131	0.4007	0.106*	
C4	0.17463 (9)	1.22525 (19)	0.31288 (14)	0.0805 (5)	
H4	0.1550	1.3052	0.2927	0.097*	
C5	0.15015 (7)	1.10443 (17)	0.27736 (11)	0.0634 (4)	
H5	0.1145	1.1027	0.2331	0.076*	
C6	0.17940 (6)	0.98494 (15)	0.30850 (10)	0.0515 (4)	
C7	0.16701 (6)	0.84417 (15)	0.29109 (10)	0.0515 (4)	
C8	0.21325 (6)	0.77376 (17)	0.34289 (11)	0.0633 (4)	
C9	0.22490 (9)	0.6260 (2)	0.35031 (16)	0.0933 (7)	
H9A	0.1921	0.5782	0.3126	0.140*	
H9B	0.2339	0.5977	0.4090	0.140*	
H9C	0.2562	0.6069	0.3341	0.140*	
C10	0.11288 (6)	0.78539 (15)	0.23107 (9)	0.0505 (3)	
C11	0.09375 (6)	0.81897 (15)	0.13777 (9)	0.0504 (3)	
C12	0.03693 (6)	0.81403 (17)	0.08275 (10)	0.0597 (4)	
H12	0.0100	0.7934	0.1054	0.072*	
C13	0.02008 (7)	0.83903 (18)	−0.00419 (10)	0.0634 (4)	
H13	−0.0181	0.8348	−0.0391	0.076*	
C14	0.05803 (7)	0.87039 (16)	−0.04178 (10)	0.0596 (4)	
C15	0.11407 (7)	0.87811 (17)	0.01237 (11)	0.0625 (4)	
H15	0.1407	0.8999	−0.0106	0.075*	
C16	0.13152 (6)	0.85409 (16)	0.10024 (10)	0.0595 (4)	
H16	0.1696	0.8617	0.1352	0.071*	
C17	0.03825 (9)	0.8940 (2)	−0.13756 (12)	0.0831 (6)	
H17A	0.0121	0.8244	−0.1679	0.125*	
H17B	0.0700	0.8925	−0.1543	0.125*	
H17C	0.0199	0.9802	−0.1517	0.125*	
C18	0.08264 (6)	0.70194 (16)	0.25982 (10)	0.0565 (4)	
H18	0.0523	0.6599	0.2169	0.068*	
C19	0.09043 (6)	0.66727 (15)	0.34913 (10)	0.0528 (4)	
C20	0.11046 (6)	0.75841 (15)	0.41800 (10)	0.0543 (4)	
H20	0.1225	0.8435	0.4090	0.065*	
C21	0.11272 (6)	0.72415 (17)	0.49952 (10)	0.0590 (4)	
H21	0.1254	0.7874	0.5439	0.071*	
C22	0.09638 (7)	0.59720 (19)	0.51636 (11)	0.0676 (5)	
C23	0.07698 (9)	0.50732 (18)	0.44865 (13)	0.0774 (5)	
H23	0.0658	0.4216	0.4582	0.093*	
C24	0.07365 (7)	0.54136 (17)	0.36668 (12)	0.0682 (5)	
H24	0.0598	0.4784	0.3223	0.082*	
C25	0.09838 (12)	0.5605 (3)	0.60507 (14)	0.1054 (8)	
H25A	0.0689	0.4971	0.5994	0.158*	
H25B	0.0932	0.6403	0.6337	0.158*	
H25C	0.1343	0.5208	0.6385	0.158*	
N1	0.25296 (6)	0.86401 (15)	0.39323 (10)	0.0757 (4)	
H1	0.2854	0.8423	0.4314	0.091*	

### Atomic displacement parameters (Å^2^)

**Table:**

	*U*^11^	*U*^22^	*U*^33^	*U*^12^	*U*^13^	*U*^23^
C1	0.0462 (8)	0.0636 (11)	0.0714 (11)	−0.0050 (7)	0.0125 (8)	−0.0026 (8)
C2	0.0596 (10)	0.0781 (13)	0.0916 (14)	−0.0180 (10)	0.0099 (10)	−0.0150 (11)
C3	0.0817 (13)	0.0645 (12)	0.1094 (16)	−0.0190 (10)	0.0276 (12)	−0.0165 (11)
C4	0.0811 (13)	0.0534 (10)	0.1020 (15)	−0.0002 (9)	0.0296 (11)	0.0019 (10)
C5	0.0550 (9)	0.0580 (10)	0.0717 (11)	0.0009 (7)	0.0182 (8)	0.0045 (8)
C6	0.0427 (7)	0.0534 (9)	0.0565 (8)	−0.0040 (6)	0.0171 (6)	0.0002 (7)
C7	0.0410 (7)	0.0523 (8)	0.0582 (9)	−0.0013 (6)	0.0160 (6)	−0.0001 (7)
C8	0.0474 (8)	0.0578 (10)	0.0757 (11)	0.0028 (7)	0.0137 (8)	0.0006 (8)
C9	0.0737 (12)	0.0647 (12)	0.1225 (18)	0.0173 (10)	0.0170 (12)	0.0056 (11)
C10	0.0429 (7)	0.0502 (8)	0.0553 (8)	0.0000 (6)	0.0156 (6)	−0.0034 (7)
C11	0.0458 (7)	0.0480 (8)	0.0558 (9)	0.0010 (6)	0.0177 (7)	−0.0023 (7)
C12	0.0464 (8)	0.0695 (10)	0.0618 (10)	−0.0015 (7)	0.0193 (7)	0.0032 (8)
C13	0.0464 (8)	0.0732 (11)	0.0616 (10)	−0.0019 (8)	0.0110 (7)	0.0007 (8)
C14	0.0624 (9)	0.0567 (9)	0.0572 (9)	−0.0003 (7)	0.0203 (8)	−0.0041 (7)
C15	0.0579 (9)	0.0693 (11)	0.0655 (10)	−0.0033 (8)	0.0295 (8)	−0.0004 (8)
C16	0.0451 (8)	0.0663 (10)	0.0631 (10)	−0.0035 (7)	0.0162 (7)	−0.0028 (8)
C17	0.0852 (13)	0.1020 (15)	0.0579 (11)	−0.0040 (11)	0.0230 (10)	0.0002 (10)
C18	0.0494 (8)	0.0578 (9)	0.0570 (9)	−0.0073 (7)	0.0147 (7)	−0.0072 (7)
C19	0.0450 (8)	0.0514 (8)	0.0585 (9)	−0.0012 (6)	0.0160 (7)	0.0020 (7)
C20	0.0488 (8)	0.0496 (8)	0.0610 (9)	−0.0002 (6)	0.0173 (7)	0.0024 (7)
C21	0.0514 (8)	0.0613 (10)	0.0563 (9)	0.0053 (7)	0.0121 (7)	0.0024 (7)
C22	0.0667 (10)	0.0669 (11)	0.0645 (11)	0.0082 (8)	0.0202 (8)	0.0165 (9)
C23	0.0913 (13)	0.0547 (10)	0.0850 (13)	−0.0062 (9)	0.0326 (11)	0.0148 (9)
C24	0.0755 (11)	0.0527 (10)	0.0722 (11)	−0.0104 (8)	0.0239 (9)	−0.0031 (8)
C25	0.136 (2)	0.1029 (17)	0.0779 (14)	0.0039 (15)	0.0424 (14)	0.0278 (12)
N1	0.0441 (7)	0.0726 (10)	0.0869 (11)	0.0043 (7)	−0.0010 (7)	0.0016 (8)

### Geometric parameters (Å, º)

**Table:**

C1—N1	1.367 (2)	C13—H13	0.9300
C1—C2	1.387 (2)	C14—C15	1.376 (2)
C1—C6	1.403 (2)	C14—C17	1.502 (2)
C2—C3	1.364 (3)	C15—C16	1.383 (2)
C2—H2	0.9300	C15—H15	0.9300
C3—C4	1.380 (3)	C16—H16	0.9300
C3—H3	0.9300	C17—H17A	0.9600
C4—C5	1.377 (2)	C17—H17B	0.9600
C4—H4	0.9300	C17—H17C	0.9600
C5—C6	1.392 (2)	C18—C19	1.470 (2)
C5—H5	0.9300	C18—H18	0.9300
C6—C7	1.436 (2)	C19—C24	1.388 (2)
C7—C8	1.362 (2)	C19—C20	1.398 (2)
C7—C10	1.485 (2)	C20—C21	1.386 (2)
C8—N1	1.374 (2)	C20—H20	0.9300
C8—C9	1.491 (3)	C21—C22	1.389 (2)
C9—H9A	0.9600	C21—H21	0.9300
C9—H9B	0.9600	C22—C23	1.375 (3)
C9—H9C	0.9600	C22—C25	1.510 (3)
C10—C18	1.344 (2)	C23—C24	1.383 (2)
C10—C11	1.483 (2)	C23—H23	0.9300
C11—C16	1.388 (2)	C24—H24	0.9300
C11—C12	1.395 (2)	C25—H25A	0.9600
C12—C13	1.372 (2)	C25—H25B	0.9600
C12—H12	0.9300	C25—H25C	0.9600
C13—C14	1.384 (2)	N1—H1	0.8600
			
N1—C1—C2	130.67 (16)	C13—C14—C17	120.83 (15)
N1—C1—C6	106.90 (14)	C14—C15—C16	121.29 (15)
C2—C1—C6	122.41 (16)	C14—C15—H15	119.4
C3—C2—C1	117.58 (17)	C16—C15—H15	119.4
C3—C2—H2	121.2	C15—C16—C11	121.84 (14)
C1—C2—H2	121.2	C15—C16—H16	119.1
C2—C3—C4	121.40 (18)	C11—C16—H16	119.1
C2—C3—H3	119.3	C14—C17—H17A	109.5
C4—C3—H3	119.3	C14—C17—H17B	109.5
C5—C4—C3	121.23 (18)	H17A—C17—H17B	109.5
C5—C4—H4	119.4	C14—C17—H17C	109.5
C3—C4—H4	119.4	H17A—C17—H17C	109.5
C4—C5—C6	119.17 (16)	H17B—C17—H17C	109.5
C4—C5—H5	120.4	C10—C18—C19	129.53 (14)
C6—C5—H5	120.4	C10—C18—H18	115.2
C5—C6—C1	118.19 (14)	C19—C18—H18	115.2
C5—C6—C7	134.86 (14)	C24—C19—C20	116.88 (15)
C1—C6—C7	106.93 (13)	C24—C19—C18	119.56 (14)
C8—C7—C6	107.33 (13)	C20—C19—C18	123.40 (14)
C8—C7—C10	126.10 (14)	C21—C20—C19	121.10 (15)
C6—C7—C10	126.51 (13)	C21—C20—H20	119.4
C7—C8—N1	108.43 (15)	C19—C20—H20	119.4
C7—C8—C9	130.86 (16)	C20—C21—C22	121.36 (16)
N1—C8—C9	120.72 (15)	C20—C21—H21	119.3
C8—C9—H9A	109.5	C22—C21—H21	119.3
C8—C9—H9B	109.5	C23—C22—C21	117.47 (16)
H9A—C9—H9B	109.5	C23—C22—C25	121.24 (18)
C8—C9—H9C	109.5	C21—C22—C25	121.27 (18)
H9A—C9—H9C	109.5	C22—C23—C24	121.59 (17)
H9B—C9—H9C	109.5	C22—C23—H23	119.2
C18—C10—C11	120.81 (13)	C24—C23—H23	119.2
C18—C10—C7	121.24 (14)	C23—C24—C19	121.57 (16)
C11—C10—C7	117.94 (12)	C23—C24—H24	119.2
C16—C11—C12	116.48 (14)	C19—C24—H24	119.2
C16—C11—C10	121.80 (13)	C22—C25—H25A	109.5
C12—C11—C10	121.71 (13)	C22—C25—H25B	109.5
C13—C12—C11	121.11 (14)	H25A—C25—H25B	109.5
C13—C12—H12	119.4	C22—C25—H25C	109.5
C11—C12—H12	119.4	H25A—C25—H25C	109.5
C12—C13—C14	122.23 (14)	H25B—C25—H25C	109.5
C12—C13—H13	118.9	C1—N1—C8	110.39 (13)
C14—C13—H13	118.9	C1—N1—H1	124.8
C15—C14—C13	117.00 (15)	C8—N1—H1	124.8
C15—C14—C17	122.17 (15)		
			
N1—C1—C2—C3	−178.7 (2)	C10—C11—C12—C13	−176.84 (15)
C6—C1—C2—C3	−0.5 (3)	C11—C12—C13—C14	−0.1 (3)
C1—C2—C3—C4	−0.2 (3)	C12—C13—C14—C15	−1.2 (3)
C2—C3—C4—C5	0.1 (3)	C12—C13—C14—C17	178.54 (17)
C3—C4—C5—C6	0.7 (3)	C13—C14—C15—C16	0.7 (2)
C4—C5—C6—C1	−1.4 (2)	C17—C14—C15—C16	−179.04 (16)
C4—C5—C6—C7	176.60 (17)	C14—C15—C16—C11	1.1 (3)
N1—C1—C6—C5	179.83 (14)	C12—C11—C16—C15	−2.3 (2)
C2—C1—C6—C5	1.3 (3)	C10—C11—C16—C15	176.34 (15)
N1—C1—C6—C7	1.32 (18)	C11—C10—C18—C19	−172.65 (14)
C2—C1—C6—C7	−177.21 (16)	C7—C10—C18—C19	8.8 (2)
C5—C6—C7—C8	179.97 (17)	C10—C18—C19—C24	−150.56 (17)
C1—C6—C7—C8	−1.89 (18)	C10—C18—C19—C20	34.2 (2)
C5—C6—C7—C10	−2.8 (3)	C24—C19—C20—C21	−0.7 (2)
C1—C6—C7—C10	175.31 (14)	C18—C19—C20—C21	174.69 (14)
C6—C7—C8—N1	1.72 (19)	C19—C20—C21—C22	1.4 (2)
C10—C7—C8—N1	−175.50 (14)	C20—C21—C22—C23	−1.0 (2)
C6—C7—C8—C9	−177.8 (2)	C20—C21—C22—C25	−179.43 (17)
C10—C7—C8—C9	5.0 (3)	C21—C22—C23—C24	−0.1 (3)
C8—C7—C10—C18	58.0 (2)	C25—C22—C23—C24	178.30 (19)
C6—C7—C10—C18	−118.71 (18)	C22—C23—C24—C19	0.9 (3)
C8—C7—C10—C11	−120.58 (17)	C20—C19—C24—C23	−0.5 (2)
C6—C7—C10—C11	62.7 (2)	C18—C19—C24—C23	−176.00 (16)
C18—C10—C11—C16	−152.49 (15)	C2—C1—N1—C8	178.08 (19)
C7—C10—C11—C16	26.1 (2)	C6—C1—N1—C8	−0.3 (2)
C18—C10—C11—C12	26.1 (2)	C7—C8—N1—C1	−0.9 (2)
C7—C10—C11—C12	−155.35 (14)	C9—C8—N1—C1	178.67 (18)
C16—C11—C12—C13	1.8 (2)		
